# TGF-β Sustains Tumor Progression through Biochemical and Mechanical Signal Transduction

**DOI:** 10.3390/cancers10060199

**Published:** 2018-06-14

**Authors:** Robert L. Furler, Douglas F. Nixon, Christine A. Brantner, Anastas Popratiloff, Christel H. Uittenbogaart

**Affiliations:** 1 Department of Medicine, Division of Infectious Diseases, Weill Cornell Medicine, 413 E 69th St., Belfer Research Building, New York, NY 10021, USA; dnixon@med.cornell.edu; 2 GW Nanofabrication and Imaging Center, Office of the Vice President for Research, George Washington University, Washington, DC 20052, USA; chrisbrantner@gwu.edu (C.A.B.); anastas@gwu.edu (A.P.); 3 Departments of Microbiology, Immunology and Molecular Genetics, Medicine, Pediatrics, UCLA AIDS Institute and the Jonsson Comprehensive Cancer Center, University of California, 615 Charles E. Young Drive South, BSRB2, Los Angeles, CA 90095, USA; uittenbo@ucla.edu

**Keywords:** TGF-β, cancer, immunosuppression, TAK1, mechanobiology, extracellular matrix, tensegrity, DNA damage

## Abstract

Transforming growth factor β (TGF-β) signaling transduces immunosuppressive biochemical and mechanical signals in the tumor microenvironment. In addition to canonical SMAD transcription factor signaling, TGF-β can promote tumor growth and survival by inhibiting proinflammatory signaling and extracellular matrix (ECM) remodeling. In this article, we review how TGF-β activated kinase 1 (TAK1) activation lies at the intersection of proinflammatory signaling by immune receptors and anti-inflammatory signaling by TGF-β receptors. Additionally, we discuss the role of TGF-β in the mechanobiology of cancer. Understanding how TGF-β dampens proinflammatory responses and induces pro-survival mechanical signals throughout cancer development is critical for designing therapeutics that inhibit tumor progression while bolstering the immune response.

## 1. Introduction

Transforming growth factor β (TGF-β) signaling can dampen immune responses during cancer progression through biochemical and mechanical signal transduction pathways. Because TGF-β receptors (TGF-βR’s) are found on several cell types, tumor-derived TGF-β can create a pro-tumorigenic microenvironment by influencing the activity of surrounding leukocytes, endothelial cells, and fibroblasts. The TGF-β superfamily consists of at least 33 genes [1], which are often grouped into either the TGF-β-like family (TGF-β, activin, inhibin, nodal, and lefty) and the bone morphogenetic protein (BMP)-like family (BMP, Growth Differentiation Factor (GDF), Anti-Müllerian Hormone (AMH), and Müllerian Inhibiting Substance (MIS)) [2,3]. Downstream from these receptors, TGF-β can activate SMAD-dependent and -independent biochemical pathways that promote tumor growth and suppress the immune system [4]. However, these pathways are not constitutively active. TGF-β is commonly expressed in a latent form and is activated following extracellular matrix (ECM) remodeling. Subsequent TGF-β signaling increases the production of new ECM components. This homeostatic feedback loop is critical for cancer growth. The ECM found within the tumor microenvironment shapes cancer mechanobiology by simultaneously providing growth signals to the tumor cell while suppressing the immune response.

Despite its well-known immunosuppressive capabilities, TGF-β signaling has been shown to have contrary effects on tumor growth during disease progression [5,6,7]. TGF-β family members display anti- and pro-tumorigenic properties depending on the stage of tumor progression [8,9,10,11].

Early in disease progression, TGF-β appears to play an anti-tumorigenic role by hindering tumor proliferation and metastasis. For example, in early stages of breast cancer, the TGF-β family member BMP7 represses human telomerase reverse transcriptase (hTERT) through a BMP Receptor II- and SMAD3-dependent manner. Chronic exposure of cancer cells to BMP7 has been shown to induce the shortening of cancer cell telomeres and subsequent apoptosis [12]. TGF-β members can also act on surrounding cells as cancer-associated fibroblasts to inhibit tumor progression and metastasis at early stages of disease [13].

In contrast, TGF-β signaling takes on a pro-tumorigenic response in later stages of disease. Elevated levels of TGF-β1 in advanced-stage breast cancers were associated with tumor size, decreased tumor cell differentiation, epithelial to mesenchymal transition (EMT), and increased metastasis to axillary lymph nodes [14,15,16,17,18]. EMT and more aggressive phenotypes of late-stage prostate cancers were also associated with elevated TGF-β1 [19]. Inhibiting TGF-β1 receptors or their downstream SMAD signaling at later stages of cancer enhanced chemotherapeutic action [20,21,22] and radiation treatment effects [23,24]. Multiple TGF-β inhibitors have been evaluated in preclinical and clinical trials and have been detailed in other reviews [25].

To understand the multifaceted roles of TGF-β in cancer, we review two ways TGF-β family members promote tumor growth. TGF-β inhibits proinflammatory signaling in tumor-infiltrating leukocytes and alters the mechanobiology of the tumor microenvironment.

## 2. TGF-β Inhibits Proinflammatory Signaling in Tumor-Infiltrating Leukocytes

**Figure 1:**
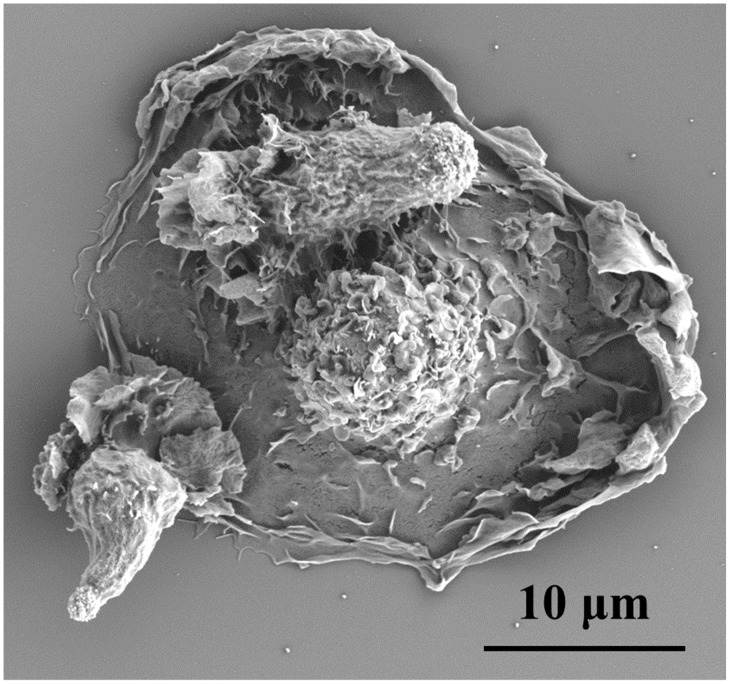
T cells and macrophages exhibit immunosuppressive qualities in tumor microenvironments. Despite presence of macrophages (larger egg-like cell in scanning electron microscopy image taken by our group) and T cells (two smaller cells scanning the surface of the macrophage), transforming growth factor β1 (TGF-β1) in the tumor microenvironment inhibited proinflammatory signaling in these leukocytes.

Tumor-infiltrating leukocytes can both express and respond to TGF-β. Signaling through TGF-βR’s can inhibit leukocyte proliferation, differentiation, and survival [1,26,27,28,29]. These effects can be reversed in leukocytes such as macrophages and T cells following the inhibition of TGF-β signaling [30,31]. Macrophages and T cells (Figure 1) can both produce and respond to TGF-β in the tumor microenvironment.

Tumor-associated macrophages often exhibit an immunosuppressive M2 phenotype by expressing interleukin 10 (IL-10), arginase-1, and TGF-β1 [32]. TGF-β1 can further inhibit expression of the proinflammatory genes inducible nitric oxide synthase (INOS) and matrix metalloproteinase 12 (MMP-12) in these macrophages [33]. Macrophage-derived TGF-β was also shown to enhance EMT in hepatocellular carcinoma [34] and metastasis in non-small-cell lung cancer [35].

In addition to macrophages, T cells also exhibit immunosuppressive phenotypes in cancer. Despite the presence of tumor antigen-specific T cells in the tumor microenvironment, these cells usually express markers of anergy and senescence [36,37]. Elevated TGF-β1 levels were shown to be associated with increased CD4+CD25+FoxP3+ regulatory T cells (Treg), EMT, and more aggressive phenotypes in prostate cancers [19,38]. TGF-β1 also induces Treg, Natural Killer T (NKT) cells, and Type 1 regulatory T cell (Tr1) [38,39,40,41,42] and inhibits proinflammatory signaling in T cells [23]. TGF-β can inhibit several proinflammatory signaling cascades in these leukocytes, but we focus on the role of TGF-β in inhibiting proinflammatory biochemical pathways that induce TGF-β activated kinase 1 (TAK1) activation.

As a tumor grows and metastasizes, there is significant tissue damage, which causes the release of proinflammatory cytokines that recruit leukocytes. Proinflammatory cytokines such as interleukin 1β (IL-1β) and tumor necrosis factor alpha (TNF-α) signal through transmembrane receptors to alert surrounding cells of homeostatic stress. Along with the IL-1 receptor (IL-1R) and TNF receptors (TNFRs), proinflammatory signals can be transmitted to leukocytes through Toll-like receptors (TLRs) and antigen-specific receptors on T cells (TCRs) and B cells (BCRs). The immunosuppressive capabilities of TGF-β have been extensively studied as an external pressure in the cancer setting. TGF-β can dampen proinflammatory signals within infiltrating leukocytes via downstream transcription factors called SMADs or through SMAD-independent pathways [4].

**Figure 2:**
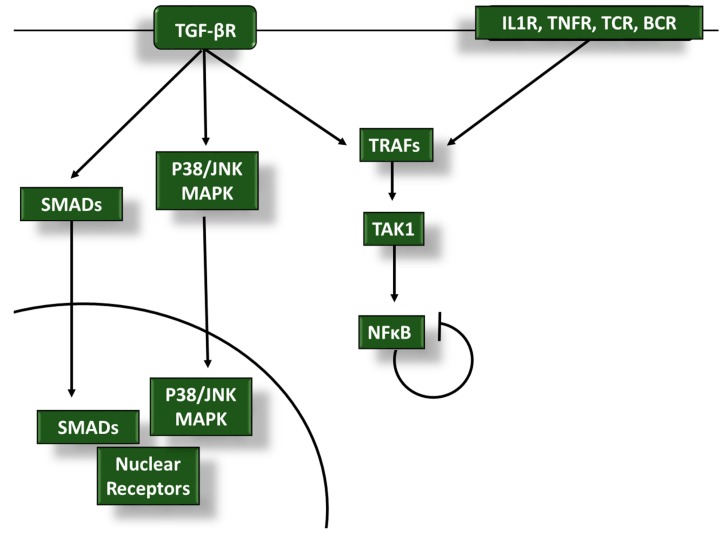
Transforming growth factor β (TGF-β) dampens anti-tumor proinflammatory signaling in infiltrating leukocytes through TGF-β activated kinase 1 (TAK1), nuclear factor kappa-light-chain-enhancer of activated B cells (NFκB), and mitogen-activated protein kinase (MAPK) modulation: tumor necrosis factor receptor (TNFR)-associated factor (TRAF) and TAK1 activity is regulated by the anti-inflammatory TGF-β receptor (TGF-βR) and proinflammatory interleukin 1 receptor (IL-1R), TNFRs, T cell receptors (TCRs), and B cell receptors (BCRs). TGF-β signaling interferes with TRAF and TAK1 activation to alter NFκB signaling in tumor-associated leukocytes to blunt immune responses during cancer progression. TGF-β modulation of the p38/JNK MAPK pathways and SMAD activity also play a role in inhibiting proinflammatory signals.

One SMAD-independent pathway that inhibits inflammation is the TGF-β-mediated modulation of TNFR-associated factor (TRAF) and TAK1 signaling, which lies at an intersection with proinflammatory signaling downstream from IL-1R, TNFRs, and TLRs, as well as the TCRs and BCRs (Figure 2). These diverse receptors use TRAF mediators to converge on the activation of TAK1 to stimulate the nuclear factor kappa-light-chain-enhancer of activated B cells (NFκB) and c-Jun N-terminal kinase (JNK)/p38 mitogen-activated protein kinase (MAPK) pathways [43,44,45]. The JNK and p38 stress pathways induce effectors that eliminate the extracellular stress; however, prolonged activation of these pathways can induce apoptosis [44]. The concomitant NFκB activation maintains anti-apoptotic signals until the stress is resolved. TGF-β1, and other TGF-β family members such as BMP, also influence TRAF signaling to alter the activity of TAK1 and downstream NFκB/JNK/p38 signaling [46,47,48] and promote tumor survival and metastasis. TRAF6 activation has been shown to ubiquitinate TGF-βRI and induce cleavage of the intracellular domain of the receptor, which can migrate to the nucleus to induce and interact with transcription factors such as Snail and Slug to promote EMT in cancer cells [49,50]. These transcription factors can further activate TGF-β signaling in breast cancer [51]. NFκB activation downstream from TLR4 can also elevate TGF-β signaling by inhibiting the pseudoreceptor BMP and activin membrane-bound inhibitor (BAMBI) [52,53]. The extensive cross-talk between TGF-β and proinflammatory signaling at the TRAF/TAK1/NFκB axis may be an important target for promoting an effective immune response to tumors.

## 3. TGF-β Signaling Promotes Tumor Growth and Inhibits Inflammation through Mechanobiology

In addition to inhibiting biochemical proinflammatory signaling cascades within leukocytes, TGF-β can inhibit the immune system and support tumor growth through mechanical transduction pathways. The ECM provides mechanical cues to surrounding cells through mechanotransduction. ECM remodeling throughout cancer progression is critical for tumor growth, metastasis, and angiogenesis; however, extensive ECM degradation can promote inflammation and inhibit proliferation. Cancer cells alter their extracellular environment with proteases during metastasis and build collagen-rich microenvironments at new loci of proliferation. TGF-β signaling plays a large role in the ECM and fibrosis seen within the tumor microenvironment. Collagen, fibronectin, and other ECM proteins can provide pro-tumor growth signals at the same time as providing a physical barrier to infiltrating leukocytes.

Mechanobiology links TGF-β, cancer cell survival, and inflammation [54]. In addition to chemical cues from the environment, cells receive critical homeostatic information from the surrounding ECM and neighboring cells through forces applied to adhesion receptors. One way mechanical information can be transmitted is through transcription factors such as Yes-associated protein (YAP) and Transcriptional coactivator with PDZ binding motif protein (TAZ), which can alter cell shape and behavior [55,56,57,58]. The intersection of YAP/TAZ and TGF-β mechanotransduction signaling in late-stage cancers has been linked to tumor invasion and fibrosis [59,60,61,62].

Remodeling of the ECM throughout cancer progression influences TGF-β signaling and alters these mechanical forces. ECM-derived forces directly impact tumor growth, metastasis, and immune evasion [63]. In mechanobiology, there is a bidirectional information flow between cells and their extracellular environment, called dynamic reciprocity. This relationship between cells and their extracellular environment dictates cellular proliferation, migration, differentiation, and survival [64,65,66,67,68,69]. Mechanical information can be transduced by forces applied to physical linkages between the extracellular environment and the DNA. This mechanical conduit consists of the following:

- ECM;
- cell adhesion receptors;
- cytoskeleton;
- nuclear membrane adaptors;
- chromatin.

Many eukaryotic cells require adhesions and these other mechanical components to stimulate growth and survival. Typical cells also inhibit their proliferation in densely packed environments. However, cancer cells exhibit anchorage independence and lack density-dependent growth inhibition. The ability of tumors to overcome these mechanical checkpoints may be due to elevated levels of TGF-β signaling that strengthens dynamic reciprocity in the tumor microenvironment. We review how TGF-β signaling influences the ECM structure and nuclear mechanobiology in cancer.

### 3.1. TGF-β and ECM

**Figure 3:**
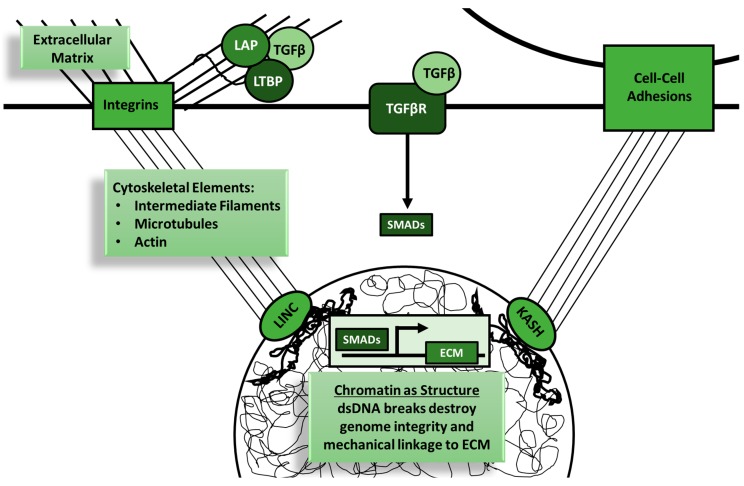
Transforming growth factor β (TGF-β) signaling is intimately linked with the mechanobiology of cells. Dynamic reciprocity is the concept of bidirectional influence of cells and their microenvironment, including adhesions to the extracellular matrix (ECM) and to surrounding cells. Physical linkages between the extracellular microenvironment are created by plasma membrane adhesion receptors, the cytoskeleton, nuclear membrane KASH (Klarsicht, ANC-1, Syne Homology)- and SUN (Sad1p, UNC-84)-domain proteins, and chromatin. KASH- and SUN-domain proteins bind to each other to form linker of nucleoskeleton and cytoskeleton (LINC) complexes that connect the cytoskeleton to the nucleoskeleton. TGF-β signaling plays an important role in this dynamic reciprocity at both the ECM and nuclear levels. Inactive TGF-β binds to latency-associated peptide (LAP) or latent TGF-β binding proteins (LTBPs) in the ECM. ECM degradation or remodeling relieves mechanical tension on the cell down to the nuclear level while simultaneously increasing the bioavailability of active TGF-β. DNA damage can also decrease the structural integrity of this mechanical tension. TGF-β signaling restores this mechanical homeostasis through upregulation of ECM components and DNA repair enzymes. Tumor cells with elevated TGF-β signaling are able to restore mechanical homeostasis despite ongoing ECM remodeling and DNA damage.

Dynamic reciprocity is exemplified by TGF-β activation and ECM homeostasis (Figure 3). TGF-β proteins are produced by several cell types and secreted in an inactive form along with latency-associated peptide (LAP), which associates with latent TGF-β binding proteins (LTBPs) [70,71]. These latent TGF-β complexes bind to ECM proteins, including fibrillins and fibronectins. TGF-β is activated when LAP or the ECM is degraded during cancer, infection, or wounding [72]. Following a mechanical cue that the ECM has been degraded, activated TGF-β family members (including TGF-β1, BMP, activins, and growth differentiation factors) signal through TGF-βR to induce expression of new ECM proteins [73,74]. After newly expressed matrix proteins are secreted, the remaining TGF-β returns to its latent state. In addition to biochemical signals derived from TGF-βR signaling, ECM degradation transmits mechanical signals to surrounding cells by releasing tension on cell adhesions. These mechanical signals are influenced by TGF-β signaling and can change the nuclear shape and gene expression in tumor cells [54,75,76].

**Figure 4:**
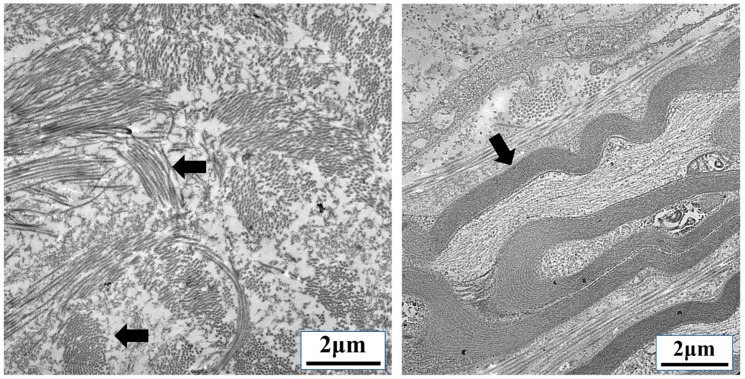
Extracellular matrix (ECM) interactions provide mechanical cues to infiltrating cells. ECM remodeling is required for tumor proliferation, metastasis, angiogenesis, and leukocyte infiltration. The image on the left shows a region with multidirectional bundles of collagen (arrows). The image on the right shows highly organized bundles of parallel collagen fibers found in body (arrow). Dense collagen networks such as these found in capsular regions of lymphoid tissue can be found within the tumor microenvironment. Collagen induced by transforming growth factor β (TGF-β) and other factors provide structural support to cancer cells and a physical barrier to leukocytes. (Transmission electron microscopy images taken by our group.)

Cancers use dynamic reciprocity to grow, metastasize, and evade the immune system by remodeling the ECM at different stages of disease progression [75,76]. The ECM consists of proteins, glycoproteins, glycosaminoglycans, and other molecules that function as adhesive substrates that promote signaling through integrins, growth factors, and mechanical cues (Figure 4).

During metastasis and angiogenesis, the ECM is degraded by tumor-derived proteases, which stimulates the release and activation of growth factors [77] and increases the bioactivity of latent TGF-β1 [78]. TGF-β signaling can also transactivate the epidermal growth factor receptor (EGFR) to promote breast cancer migration [79]. Following metastasis to a secondary site, the tumors use this bioactive TGF-β to induce expression of ECM components, including collagen, fibronectin, and tenascin C, along with ECM-related enzymes and chaperone proteins [80,81]. TGF-β produced by tumor-associated macrophages induces fibroblasts to express collagen and other ECM proteins [32,34]. This ECM remodeling creates a microenvironment amenable to tumor proliferation and immunosuppression.

The excessive buildup of ECM proteins is called fibrosis and contributes to the progressive ECM rigidity seen during cancer progression and throughout other chronic diseases [82,83,84]. In a breast cancer model, Liverani et al. showed that a more aggressive tumor cell line, MDA-MB-231, induced higher collagen content, collagen cross-linking, and increased ECM stiffness compared to the less aggressive cell line MCF-7 [85]. Progressive stiffening and reorganization of the ECM during cancer progression is also due to TGF-β-mediated effects on surrounding stromal cells including fibroblasts. Reactive stromal cells in human prostate cancers have a myofibroblast phenotype and exhibit increased collagen I production, vimentin, tenascin, and smooth muscle α-actin. Elevated TGF-β1 levels were also found in these prostatic intra-epithelial neoplasia [86]. TGF-β1 induces contraction of stromal fibroblasts and subsequent ECM strain [87]. This progressive ECM stiffness is directly correlated with tumor aggressiveness and can dampen the immune response [63,75,88,89,90].

Tissue fibrosis has several detrimental effects during chronic diseases. In addition to local disturbances in tissue function, fibrosis creates a physical barrier to infiltrating immune effectors and potential therapeutic agents. Netti et al. reported that the fibrotic ECM prevents therapeutic IgG from penetrating solid tumors [91], and others have shown that chemotherapeutic drugs may not reach fibrotic tissue in small-cell lung cancers [92].

### 3.2. TGF-β and Nuclear Mechanobiology in Cancer

**Figure 5:**
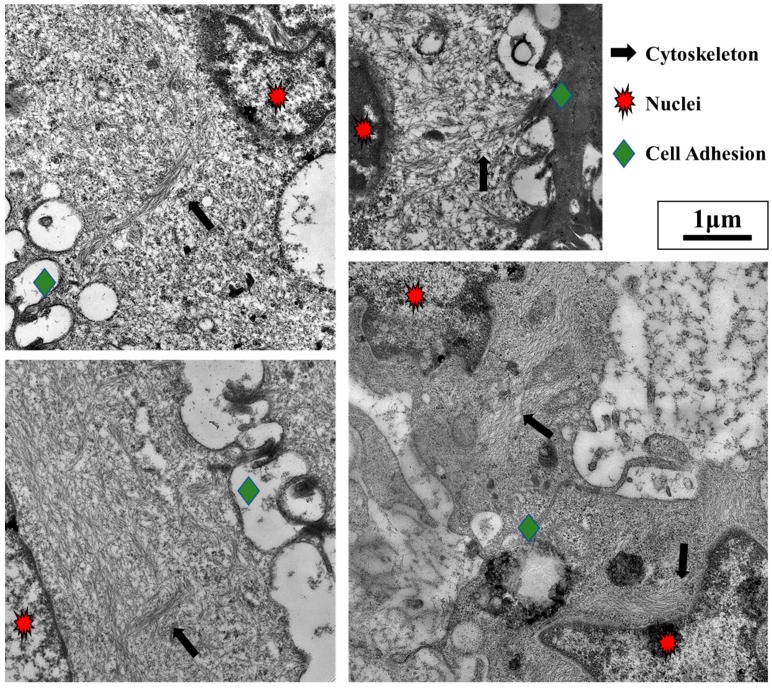
Cell adhesions to other cells and the extracellular matrix (ECM) are mechanically linked to the nucleus. Leukocytes and metastatic tumors often migrate to secondary lymphoid tissues. As seen from the lymphoid tissue images above, cells are intimately connected through several cell–cell adhesions (**green diamonds**) as they migrate and proliferate in this environment. The four images above show that cell adhesions (**green diamonds**) are directly linked to the nuclei (**red stars**) by cytoskeletal elements (**black arrows**). (Transmission electron microscopy images taken by our group).

Tensegrity is a structural concept that can be used to understand the mechanics of three-dimensional structures such as cells. Cellular structures are physically connected through cytoskeletal elements, and forces applied to one area can have effects throughout the cell [65,66,67,68,69,93,94,95,96,97]. Forces from the ECM in the tumor microenvironment can have direct impacts on gene expression in tumor and immune cells. These mechanical forces are transmitted through cellular adhesions containing receptors such as integrins, which are one of the primary transducers of mechanical information from the environment to the nucleus [93,95,97,98]. TGF-β can be activated by and alter expression of integrins during cancer progression [99,100,101,102,103]. Integrins and other cell adhesions transduce extracellular forces mechanically to the nucleus via the cytoskeleton (Figure 5). Cytoskeletal elements such as actin, microtubules, and vimentin have all been shown to play a role in TGF-β signaling and cancer [104,105]. Tumor metastasis and proliferation are both altered by TGF-β-mediated cytoskeletal changes [106,107]. The cytoskeleton attaches to the nuclear lamina and chromatin through nuclear membrane adaptor proteins called linker of nucleoskeleton and cytoskeleton (LINC) complexes and KASH (Klarsicht, ANC-1, Syne Homology)-domain proteins [108]. TGF-β signaling and some nuclear lamina proteins have been shown to have reciprocal regulation [109,110,111]. Forces emanating from the extracellular environment are transduced through these structural proteins and can alter the three-dimensional nuclear shape and directly impact gene expression [65,66,68,94,95,96,112,113,114]. TGF-β can influence the structural integrity of each of these sections of the mechanical conduit from the ECM to the nucleus. Additionally, TGF-β influences the structural integrity of chromatin and DNA.

**Figure 6:**
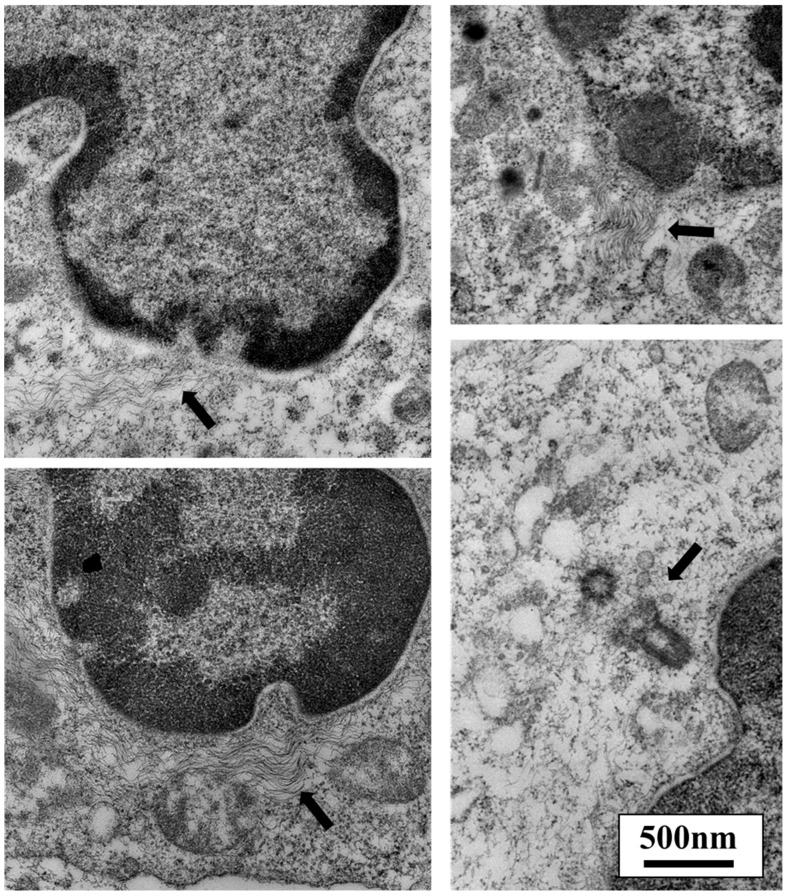
The cytoskeleton can alter nuclear shape. Cell adhesions are directly linked to the nuclear lamina and chromatin via the cytoskeleton and linker of nucleoskeleton and cytoskeleton (LINC) complexes. Forces applied to cellular adhesions have been shown to change nuclear shape and alter gene expression. Deformations at the site of cytoskeletal attachment to the nucleus can be seen in the four images above. As shown in these leukocytes, the fibrillar cytoskeletal elements are connected directly to the nucleus (arrows), primarily at sites of darkened areas of heterochromatin. Microtubules are organized at the centrosome, which contains centrioles. Microtubules are one type of cytoskeletal protein that links adhesion receptors to the nucleus. The bottom right image shows a pair of centrioles and their close proximity to the nuclear envelope (arrow). (Transmission electron microscopy images taken by our group.)

The role of mechanical forces in gene expression and the physical linkages between the cell adhesions and the chromatin (Figure 6) lead to the idea of chromatin as structural information. With the development of new technologies such as Hi-C that use chromosome conformation capturing methods [115], more emphasis is being placed on the importance of genome structure [116,117]. Genome architecture and chromosome domains are recapitulated through chromatin cross-linking followed by DNA sequencing. These approaches give a snapshot of how the genome is organized at a specific timepoint. The genomic architecture changes with time and cellular differentiation but can be influenced by TGF-β and DNA damage. SMADs have been shown to remodel the chromatin structure by binding with histone modifiers [118,119]. The integrity of the genome is also critical to maintain, exemplified by the quick repair response that is initiated following DNA damage, as prolonged DNA damage typically leads to apoptosis [120]. Maintenance of DNA structural integrity may be critical for the dynamic reciprocity between the cell and its environment through tensegrity.

Cancer cells are continuously exposed to DNA damage, yet they persist with the help of TGF-β signaling. TGF-β signaling modulates the DNA repair response and apoptosis in normal and cancerous cells. Some reports have shown that TGF-β and SMAD signaling enhances the DNA damage response to maintain genomic stability [121,122]; however, others have shown that CD44^+^/CD24^-^ cancer cells with constitutively activated TGF-β signaling are defective in DNA repair, which makes their genomes less clonal following dsDNA breaks [123]. SMADs have been shown to interact with p53 and also regulate transcription of other DNA repair enzymes [124]. Other reports indicate that TGF-β signaling aids in tumor growth by inhibiting DNA-damage-induced apoptosis. Elevated TGF-β1 signaling has been shown to confer radioresistance following double-stranded DNA damage caused by ionizing radiation [125]. TGF-β1/SMAD signaling inhibits p53 activation at both transcriptional and translational levels and subsequent apoptosis in precancerous cells [126]. Therefore, the inhibition of TGF-β1 signaling has been recommended as a possible avenue to promote cancer cell death following genotoxic stress after radiation treatment [23,24].

In addition to DNA damage and genome integrity, the structure of the nucleus changes with tumor behavior. ECM remodeling during cancer progression has been associated with nuclear deformations, subsequent gene activation, and EMT [76]. These nuclear structural changes can occur as a result of forces generated during ECM remodeling. During the early stages of cancer, the surrounding ECM can be a hindrance, as dense fibrillar ECM obstructs tumor growth, metastasis, and angiogenesis. Cancers need to degrade surrounding collagen by Matrix Metalloproteinases (MMP) and other enzymes to overcome this physical hurdle [127]. In addition to collagen, disturbances in hyalectan proteoglycans during cancer progression have been seen, along with the activation of a disintegrin-like and metalloproteinase domain with thrombospondin 1 motif (ADAMTS) enzymes [128]. The degraded matrix releases tension of the tumor cell, which can sense this mechanical signal and alter its gene expression. This mechanical stimulus may be an influential step in EMT.

**Figure 7:**
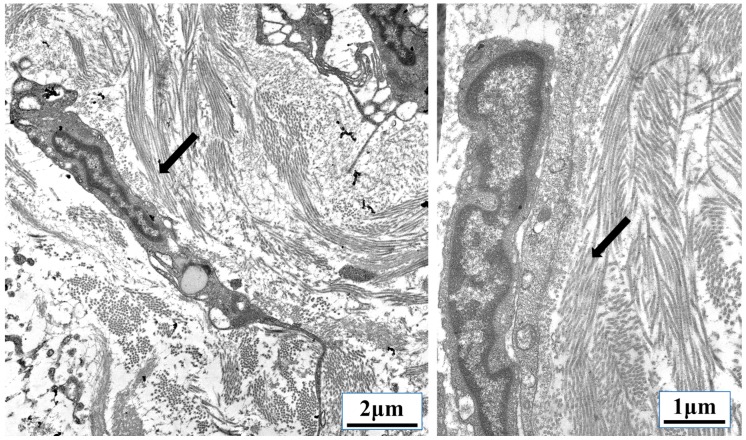
Parallel organization of extracellular matrix (ECM) fibrils can be used as migration tracts. Transforming growth factor β (TGF-β)-induced fibrosis and ECM remodeling during cancer transmits mechanical information from the tumor microenvironment directly to the nucleus of tumor cells, infiltrating leukocytes, fibroblasts, and endothelial cells. Some metastasizing cancer cells use parallel ECM fibrils, called “tumor-associated collagen signatures” (TACSs), similar to the collagen tracts (arrows) used by the leukocytes shown above migrating through lymphoid tissue. (Transmission electron microscopy images taken by our group.)

ECM stiffening during cancer progression is associated with matrix cross-linking or reorganization. In the tumor microenvironment, ECM fibrils become organized in parallel directions. These fibrils are referred to as “tumor-associated collagen signatures” (TACSs) and have been suggested to create migration routes for metastasizing cancer cells [129] such as those used by leukocytes (Figure 7). Migration through tissues with a dense ECM requires nuclear deformation and extension. This is seen in both leukocytes and cancer cells and is one more example of how the nuclear structure can be altered. The mechanical forces placed on the nucleus during migration may be critical for gene expression and cellular differentiation not only in tumor cells and leukocytes, but for all developing cells.

## 4. Materials and Methods

Scanning electron microscopy (SEM) was done on peripheral blood lymphocytes grown in RPMI growth medium supplemented with 10% fetal bovine serum (FBS), 20 IU/mL IL-2, and antibiotics. The cells were plated on fibronectin-coated coverslips for 3–7 days. Adherent cells were fixed with 2.5% glutaraldehyde, 1% paraformaldehyde, and 0.12 M sodium cacodylate buffer (pH 7.2–7.4) for 20 min at room temperature followed by 40 min of shaking on ice. Following rinsing, the cells were stained with 1% osmium tetroxide (Electron Microscopy Sciences, Hatfield, PA, USA) for 60 min on ice. The cells were dehydrated through a series of ethyl alcohol/deionized water solutions followed by critical point drying and sputter coating with iridium. Imaging was done using a FEI Teneo LV SEM instrument (Thermo Fisher Scientific, Waltham, MA, USA).

Transmission electron microscopy (TEM) was done on human lymphoid tissue. Routine EM processing was done using intact human tonsillar tissue. The tissue was fixed in 2.5% glutaraldehyde and 1% paraformaldehyde in 0.12 M sodium cacodylate buffer for 20 min at room temperature followed by 40 min of shaking on ice. The tissue was mechanically sectioned into ~1 mm^3^ tissue chunks. Cells were then stained for 1 h in 1% osmium tetroxide (Electron Microscopy Sciences, Hatfield, PA, USA). The cells were dehydrated through a series of ethyl alcohol/deionized water solutions and propylene oxide before embedding in LR White resin. Blocks were cured for 48 h at 60 °C. Polymerized blocks were trimmed, and 70 nm ultrathin sections were cut with a diamond knife on a Leica Ultramicut EM UC7 (Wetzlar, Germany) before being transferring to 200-mesh copper grids. Sections were counterstained with 1% uranyl acetate for 10 min and lead citrate for 2 min. Samples were imaged with a FEI Talos F200X-TEM (Thermo Fisher Scientific, Waltham, MA, USA) operating at an accelerating voltage of 80 kV equipped with a Ceta 4K × 4K camera.

## 5. Conclusions

The multifunctional cytokine TGF-β has been shown to have several pro-tumorigenic actions during cancer progression. TGF-β signaling can mediate immune suppression through SMAD-dependent and -independent pathways. TGF-β signaling can antagonize proinflammatory signals emanating from immune receptors (IL-1R, TLR, TNFR, TCR, and BCR) by modulating TRAF/TAK1, NFκB, and p38/JNK MAPK activation. In addition to classical biochemical signaling cascades, TGF-β in the tumor microenvironment activates pro-tumor and anti-inflammatory mechanotransduction pathways through ECM remodeling, cytoskeletal alterations, and maintenance of DNA damage. Understanding how TGF-β signaling affects proinflammatory signaling and cancer mechanobiology will be critical in designing therapeutics that inhibit tumor progression while bolstering the immune response [25].

## Glossary

Mechanotransduction: Information transmitted from the extracellular matrix (ECM) or surrounding environment to the cell via mechanical forces (e.g., tension, compression, and shear stress) [56,64,83]. Tensegrity: The cytoskeleton stabilizes cell shape through continuous tension. Cell shape is maintained by a balance of tensional forces derived from actin and intermediate filaments and their interactions with compression-resistant microtubules and cell/ECM adhesions [66,67,69,93,95,96]. Dynamic Reciprocity: A concept describing the interrelatedness of a cell and its extracellular environment. A cell receives signals from the surrounding ECM, which can influence its expression of genes involved in ECM organization and composition [129].
